# Clerical Reading

**Published:** 1861-01

**Authors:** 


**CLERICAL READING.**—To have a full appreciation of the statement about to be made, it is necessary to premise that we attend public worship where there has been no regular minister for twenty months, having different speakers, averaging almost one a week. We sit in a pew immediately in front of the pulpit, and are, perhaps, more regularly present than any other male attendant. Our custom is to read the chapter with the officiator. The locality is on Fifth Avenue, New-York. The congregation is perhaps inferior to none in our country in size, culture, liberality, and wealth, representing a capital last year of about fifty millions of dollars. For these reasons it may be inferred that a more educated class of men have been invited to officiate from time to time, and that we are a competent observer. It is perhaps safe to say, that in these twenty weeks, about fifty different men have preached for us. Out of these fifty, only two have succeeded in reading a chapter in the Bible with accuracy. All but the two have made from two to ten mistakes in reading a single chapter, mainly in omissions and interpolations; next, transpositions; third, in the indiscriminate use of the singular and plural numbers, and the addition of words of from one to three syllables. This statement is made to draw attention to the physiology of reading. To read correctly, and with the greatest ease to himself and edification of others, a man should be in good health, should have a clear brain, so as to understand thoroughly what he is reading, and the mind should be absorbed in the subject. If the stomach has been overloaded, if a hearty meal has just been eaten, or if there is any mental perturbation, any anxiety, any affectation, any straining for effect, there will be failures more or less frequent. Let our clerical readers, then, do all that is possible to enable them to go into the sacred desk, to perform the high and most responsible duties connected with their office, with presence of mind, with a due sense of their accountability, and with a most perfect abnegation of self. Let the whole soul be absorbed in the subject of the occasion, and let the service be preceded by a very moderate meal of plain food, drinking absolutely nothing but half a glass of cold water, even if that, at the said meal; for if much of any liquid is taken, it produces an uncomfortable distension of the stomach, causing a feeling of fullness and oppression, compelling the mind away from the subject and the occasion to such ignoble things as acidities, belchings, and—gas! It is most respectfully suggested that our Lenoxes, Stuarts, and McCormicks, out of their princely liberalities, found a "Reading Professorship" in some of our more prominent theological seminaries. Only think of it! it takes twenty-five educated clergymen to read a chapter in the Bible *verbatim et literatim!*

